# Simulation of Invertebrate Aggregation Shows the Importance of Stable Personality over Diversity in Consensus Decision-Making

**DOI:** 10.1371/journal.pone.0165082

**Published:** 2016-10-18

**Authors:** Mark Pogson

**Affiliations:** Department of Applied Mathematics, Liverpool John Moores University, James Parsons Building, Byrom Street, Liverpool L3 3AF, United Kingdom; Waseda University, JAPAN

## Abstract

Aggregation of many species of invertebrate is an example of a consensus decision, the success of which is central to survival. Personality is a stable form of behavioural diversity which has been observed in the aggregation process, but neither the reasons for its stability nor its effects on consensus decisions are well understood. By using an agent-based model of invertebrate aggregation, it is found that diverse personalities have only limited benefits to the experimental consensus decision-making process, but may have a more valuable role in natural settings. Importantly, although certain personalities may ostensibly have potential drawbacks at the individual level, such as choosing to rest in unfavourable places, all individuals are likely to benefit from maintaining a constant personality, which promotes group stability. These findings help to improve understanding of consensus decision-making and the prevalence of stable personality.

## Introduction

A consensus decision is a form of collective decision with the requirement of a unique outcome to which the group adheres [1,2]. Consensus decisions have many potential benefits including maintenance of group cohesion and greater speed and accuracy of outcome compared with lone decisions [3]. Success or failure of consensus decision-making can have major consequences [4]; hence its study is of fundamental importance.

Aggregation of woodlice (Crustacea: Isopoda: Oniscidea) is an example of consensus decision-making as it depends not only on shared individual preferences but also social attraction [5,6]: given a choice of two identical shelters, woodlice are observed to aggregate under just one chosen at random [7]. As the process is decentralised and self-organising it is an example of swarm intelligence [8]. Aggregation and sheltering are particularly important survival behaviours for woodlice due to their vulnerability to desiccation [9]. Similar aggregation processes exist in other invertebrates and it is likely that many of the mechanisms involved are evolutionarily conserved or convergent across species [10]. Aggregation is considered to be a precursor to more complex forms of sociality; therefore a generic description of aggregation is of wide interest [7,11].

Dynamics of invertebrates may be quite predictable at the group level, but diverse behaviour typically occurs at the individual level, which may help to increase overall exploration [2]. Diversity of individuals has previously been shown to be beneficial to group decisions, with the potential for diverse groups to outperform groups of more highly-able individuals [12,13,14,15,16]. Personality is a form of diversity where behavioural differences between individuals are temporally and contextually stable [17,18], and the shy-bold continuum is recognised as a basic component of personality in several animals [19]. The role of personality in consensus decision-making is of increasing interest [20] and has recently been observed in cockroaches [21].

The present study simulates woodlice aggregation in order to investigate the effects of individual personality on consensus decisions. While the evolutionary basis for diversity of behaviour is well established, the benefits of consistent behaviours which constitute distinct personalities are not fully understood [15,20,22,23]. The present study therefore investigates the role of diversity in woodlice aggregation and whether the stability of individual personalities is beneficial to the decision-making process compared with unstable but equally diverse individual behaviours. That is, is the benefit of individual personality that it generates diversity, or does its stability also provide advantages? By addressing this question, the aim is to improve understanding of why personality exists, why it is so prevalent across species and how it affects consensus decisions.

Existing models of invertebrate aggregation either use a compartmental differential equation approach to characterise group dynamics [21] or an agent-based approach to model explicitly the behaviour of individuals [2,11,24,25]. The present study develops an agent-based model to investigate the effects of individual personalities on decision-making at both the individual and group level. The model is evaluated against existing woodlice data due to the quality of information available in the literature, but the generic formulation is widely applicable. The present study does not aim to determine the existence of personality in woodlice, but since personality has been identified in other invertebrates with directly comparable aggregation mechanisms, woodlice are used as a model taxon in order to investigate the role of personality in consensus decision-making.

## Methods

**Fig 1:**
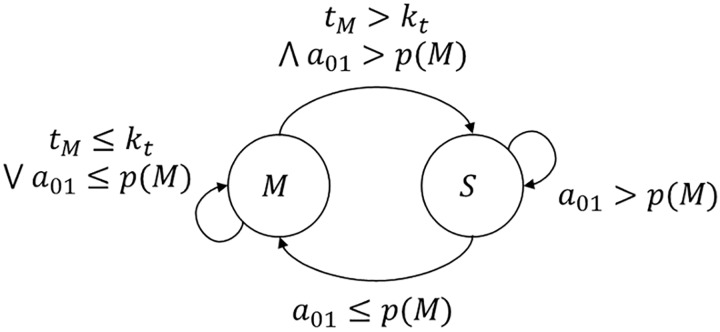
Transitions between moving state *M* and stationary state *S*. Transitions occur in discrete time. If an agent is in state *M* and the time since entering the state *t*_*M*_ does not exceed the minimum permitted time *k*_*t*_ then no transition occurs. Otherwise, a random number *a*_*01*_ is generated from a uniform distribution between 0 and 1. If *a*_*01*_ is less than the probability of moving *p*(*M*), accounting for the number of stationary neighbouring agents, the presence of shelter and personality (as shown in Eq 1), then the agent will be in state *M*; otherwise it will be in state *S*.

Each woodlouse is modelled as a distinct agent which may be either in a moving or stationary state. Space is continuous in two dimensions and time is discrete; each iteration of time is termed a time step. Transitions between the two states may occur randomly at each time step. The probability of transition depends on the presence of shelter, the number of stationary neighbouring agents, the length of time spent in the moving state and the personality of the agent, as shown in Fig 1 and discussed further below. A time-wise exponential decrease in the probability of transition between states has previously been observed at the group-level in cockroaches [26] and foraging herbivores [19], which is consistent with a fixed probability of transition occurring at regular time intervals at the individual-level, as performed here (i.e. for each additive change in time, there is a constant fractional probability of transition). It should be noted that agents only sense the local environment; there is no long-range attraction either to shelter or other agents. Agents are only attracted to adjacent stationary agents, and not moving agents, hence there is no herding behaviour. Agents do not carry out any form of learning and the group is non-hierarchical.

Agents are confined to a circular arena which contains two identical shelters on opposite sides, based on the experimental arrangement of Devigne et al. [7]. Each agent is initialised in a stationary state near the centre of the arena (which is not under shelter) with a random initial direction. When in a moving state, agents move with constant speed. An agent does not change direction until it encounters the arena boundary; the direction of the agent is then rotated such that it remains inside the boundary without changing speed (the change in direction is calculated to keep the agent within the arena, rather than being random). For simplicity, agents pass freely over one another. This is reasonable given that woodlice stack over each other in aggregates and are observed when moving to pass each other without significant change in overall direction [7]; note that agents nevertheless sense each other in terms of their behaviour, as described below. For simplicity also, the direction of movement of each agent does not fluctuate; hence once an agent encounters the boundary it will subsequently move along it due to the successive rotations required to remain within the boundary. Such behaviour is in broad agreement with experimental observations [7,27]. Since shelters are located at the edge of the arena, agents encounter them as part of their movement. Please see S1 Fig for a video of the simulated movement of agents, which also shows the arrangement of the arena and shelters.

There are five model parameters which affect agent behaviour, the combination of which gives the probability of transition between moving and stationary states at each time step, as shown in Fig 1:

1. The probability *p*_*e*_ of moving when not under shelter, neglecting sociality.
2. The probability *p*_*h*_ of moving when under shelter, neglecting sociality.
3. The change in probability of movement *f*_*g*_ due to each stationary neighbouring agent (i.e. sociality).
4. The minimum permitted duration of movement *k*_*t*_.
5. The change in probability of movement *f*_*r*_ due to personality.

The level of activity of woodlice under different environmental conditions is represented by *p*_*e*_ and *p*_*h*_ [9]. The exact value of both depends on the size of the time step, but specifically *p*_*e*_ depends on the conditions outside shelter and *p*_*h*_ depends on the quality of shelter. As woodlice have an individual preference to rest under shelter, it follows that *p*_*e*_ > *p*_*h*_.

Agents are defined as neighbouring if they are within a specified distance of each other. Parameter *f*_*g*_ modifies *p*_*e*_ and *p*_*h*_ by a constant multiplicative factor for each stationary neighbouring agent. Setting *f*_*g*_ between 0 and 1 therefore promotes aggregation as it reduces the probability of movement in the presence of stationary neighbours. A multiplicative (rather than additive) factor is used to ensure that resultant probabilities remain between 0 and 1. As agents pass freely over one another, each agent may have any number of stationary neighbours. A previous agent-based model of cockroaches imposed a limit of three neighbours that could be sensed [26]. No upper limit is used in the present model as stacking of aggregated woodlice is likely to occur; it is also believed that woodlice are attracted by pheromones in faeces, which would increase the number of influential neighbours in an aggregate [9].

Parameter *k*_*t*_ is introduced in order to allow agents to escape a stable aggregate. It is required due to the stochasticity of the model: if an agent is in a stable aggregate but starts to move, then at the next time step it would likely still be under very similar conditions which are not conducive to movement, and hence without *k*_*t*_ the agent would likely stop moving. The parameter therefore allows agents to act out decisions to leave an aggregate.

Personality is introduced to the model by modifying the probability of movement by a constant additive term *f*_*r*_ which is different for each agent. This represents the shy-bold continuum, where bold individuals are more likely to explore the environment and hence have a higher probability of moving (note the distinction from gregariousness). An additive (rather than multiplicative) term is used to produce a symmetrical effect on the probability of movement, which therefore does not alter the average behaviour of the group. Although this theoretically may generate probabilities less than 0 or greater than 1, in practice only small additive terms are considered, which would require extremely small or large pre-existing probabilities at each time step for the range to be exceeded; if this occurs, they are limited appropriately. Assumptions about the nature of personality are addressed further in the Discussion, and consideration of a multiplicative personality factor is addressed in S2 Fig.

By combining the above parameters, the probability *p*(*M*) of an agent moving at each time step is:

$$p\left(M\right)=p_{i}f_{g}{}^{n}+f_{r}$$ (1)

where *n* is the number of neighbouring stationary agents and *p*_*i*_ is the non-social probability of moving, which is equal to *p*_*h*_ when under shelter and *p*_*e*_ when not under shelter. Eq (1) does not apply if an agent is in the moving state and has been moving for less than time *k*_*t*_, in which case *p*(*M*) = 1, as described above. At each time step, the behaviour of each agent is calculated in turn; to avoid any bias due to the sequence of calculations, the order is randomised at each time step.

Planas-Sitjà et al. [21] defined the individual resting time (IRT) as a metric for personality in cockroaches; this is the length of time an individual spends under shelter. Individual (i.e. non-social) preference would be for high IRT values within the context of the experiment. For generality, fractional IRT is used in the present study; it is obtained by dividing the IRT by the total experimental time.

The shelter which contains the most agents at the end of each experiment is termed the winning shelter; the other is the losing shelter. As the two shelters are identical, both shelters initially have an equal chance of becoming the winning shelter. The winning proportion (WP) is defined as the proportion of agents under the winning shelter at the end of each experiment; it is a measure of the strength of the consensus decision.

A sensitivity analysis is performed in order to investigate the performance of the model and identify appropriate parameter values in the absence of different agent personalities (i.e. *f*_*r*_ = 0). Multiple random samples of values for the different model parameters are obtained in order to investigate a large parameter space [28], and a separate simulation is run for each combination of parameter values. The mean fractional IRT of agents is obtained for each simulation and is plotted against the corresponding value of each of the parameters. This shows the sensitivity of model results to changes in each of the parameters, concurrently accounting for the effects of all other parameters.

Using appropriate parameter values identified from the sensitivity analysis, results are obtained for aggregation dynamics with the assumption of no difference in personality between agents (i.e. *f*_*r*_ = 0). In order to evaluate the model, results are compared against equivalent experimental studies by Devigne et al. [7] and Broly et al. [5]. Given that previous models of invertebrate aggregation have accurately described aggregation dynamics in the absence of distinct personalities (including for cockroaches, in which personality has been observed), it is expected that these results should compare favourably with experimental values.

The potential role of personality in the aggregation process is subsequently investigated by measuring the effect of different values of *f*_*r*_ on IRT and WP values. This enables consideration of the costs and benefits associated with different personalities, both to individuals and the group. In light of the potential costs of certain personalities to individuals, and since previous studies have identified the importance of diversity to many forms of collective decision-making, the present study considers the effect of changeable behaviours (herein termed unstable diversity, which is obtained by non-constant values of *f*_*r*_ for each agent; this is also termed personality-related plasticity), both on the group and individuals. As opposed to the constancy of personality, this would provide diversity while potentially sharing the burden of behaviours which may ostensibly be individually undesirable but beneficial to the group (and vice versa). The consequences of this are evaluated, which provides information on the role of stable personality.

The model is programmed in Python. A time step of 1s is used in all simulations. In the moving state, agents move with speed 10mm/s, in approximate agreement with a video of woodlouse movement presented by Devigne et al. [7]; however, since movement is relative to the size of the time step, and parameter values are estimated in accordance with the size of the time step, precision of the speed value is relatively unimportant to the findings. Please see the simulation video in S1 Fig for comparison with the experimental video of Devigne et al. [7]. Woodlice are able to detect stationary neighbours within a centre-to-centre distance of 5mm. The arena radius is 100mm. Each shelter is 17.5mm in radius and touches the arena boundary, as shown in S1 Fig. For the purpose of counting the number of agents under each shelter to obtain WP (as opposed to determining whether an agent is under shelter to calculate its movement probability and IRT), an agent is included if it is within 25mm of the shelter boundary, which allows for possible spreading of aggregates beyond the shelter, as described by Devigne et al. [7]. Experiments are 45 minutes in simulated duration and 40 agents are present in each experiment. Except for the sensitivity analysis, 50 repetitions of each experiment are simulated. Parameter values are reported with results.

## Results

**Fig 2:**
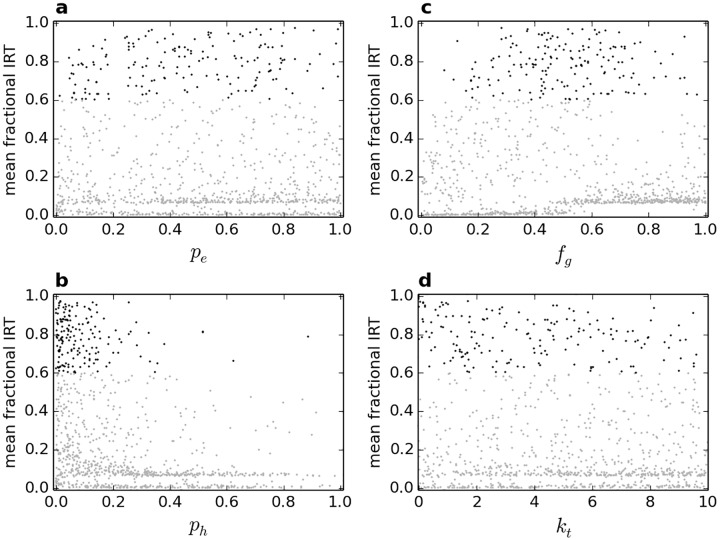
Sensitivity analysis of agent parameters with *f*_*r*_ = 0. Mean fractional IRT is the mean fraction of time agents spend under shelter in a single simulation. Each dot represents the result of a single simulation with randomised parameter values; black dots represent combinations of parameters that produce a mean fractional IRT greater than 0.6. IRT results are plotted against: (a) probability of moving when not under shelter, neglecting sociality; (b) probability *p*_*h*_ of moving when under shelter, neglecting sociality; (c) change in probability of movement *f*_*g*_ due to each stationary neighbouring agent (i.e. sociality); (d) minimum permitted duration of movement *k*_*t*_, in seconds.

Sensitivity analysis results are shown in Fig 2 for 1000 random combinations of parameter values. It is evident that a wide range of parameter values may produce a high IRT, with certain ranges more conducive to this. In particular, a low value of *p*_*h*_ is most likely to produce a high IRT, as this makes movement from shelter less likely. A medium value of *f*_*g*_ is also more likely to produce a high IRT; if this value is too high, woodlice are prone to aggregate outside shelter, and if too low they are prone not to aggregate at all. A similar explanation holds for the two bands of low IRT values: for the one at around IRT = 0, a small *f*_*g*_ value (in combination with other parameters) tends to hold agents in stable aggregates before they arrive at shelter, and for the other at around IRT = 0.1, a large *f*_*g*_ value (in combination with other parameters) means agents do not form stable aggregates and therefore only pass through shelter as part of their movement.

All parameters in Fig 2 are uniformly sampled across the presented ranges except for *p*_*h*_ which has the condition that *p*_*h*_ *< p*_*e*_ (i.e. agents individually prefer shelter) in each simulation, hence the distribution of sampled *p*_*h*_ values is skewed towards the lower end of the full range. Note that high IRT does not necessarily mean that all woodlice form a single aggregate; for this, *k*_*t*_ is important, as it allows woodlice to escape stable aggregates, thereby diminishing smaller aggregates at a greater rate than larger aggregates (since sociality makes movement from larger aggregates less likely).

**Fig 3:**
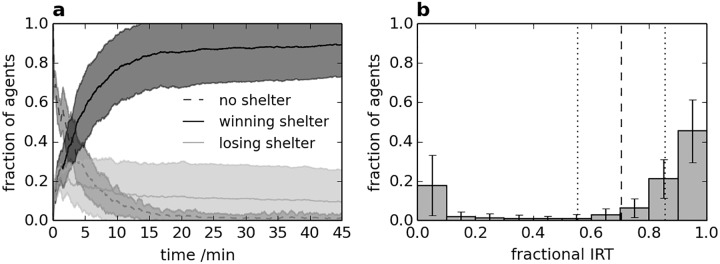
Aggregation dynamics and IRT distribution with *f*_*r*_ = 0 (i.e. no personality differences). (a) Time-series of woodlice locations. Shaded areas show the standard error of the mean from 50 repetitions of the experiment. (b) Distribution of IRT values. Error bars show the standard error of the mean for each interval. The vertical dashed line shows the mean IRT and the dotted vertical lines show the standard error.

In light of the sensitivity analysis, appropriate but arbitrary parameter values are used in all subsequent simulations: *p*_*e*_ = 0.8, *p*_*h*_ = 0.1, *f*_*g*_ = 0.5 and *k*_*t*_ = 5s. The resultant dynamics of woodlouse aggregation are shown in Fig 3, along with the distribution of IRT values. A video of part of a single simulation is included in S1 Fig.

It is evident from Fig 3a that almost all agents are under shelter at the end of each experiment: a large majority are under the winning shelter (WP ≈ 0.9) and almost all others are under the losing shelter. It should be noted that the preference to aggregate under a single shelter (i.e. the consensus decision) is an emergent behaviour of the group, arising only from the local rules and interactions of agents. Aggregation predominantly occurs within the first 15 minutes of the experiment, with relatively stable subsequent dynamics. Results are in good agreement with experimental studies by Broly et al. [5] and Devigne et al. [7] in terms of the rate, distribution and variability of aggregation. Further details are considered in the Discussion.

As shown in Fig 3b, the mean fractional IRT for the group is high, at around 0.7 of the experimental time, with a standard error (across simulations) of around 0.15. Despite no personality differences between agents, there is a wide distribution of fractional IRT values, with most agents spending nearly the entire experiment under shelter, a small number spending minimal time under shelter, and very few between the two extremes. This is due to the existence of two relatively stable equilibria for each agent: aggregation under shelter, resulting in high IRT, or aggregation outside shelter, resulting in low IRT. Aggregation outside shelter represents a trade-off between individual and social preference, which was observed by Devigne et al. [7], although Broly et al. [5] found it less significant.

**Fig 4:**
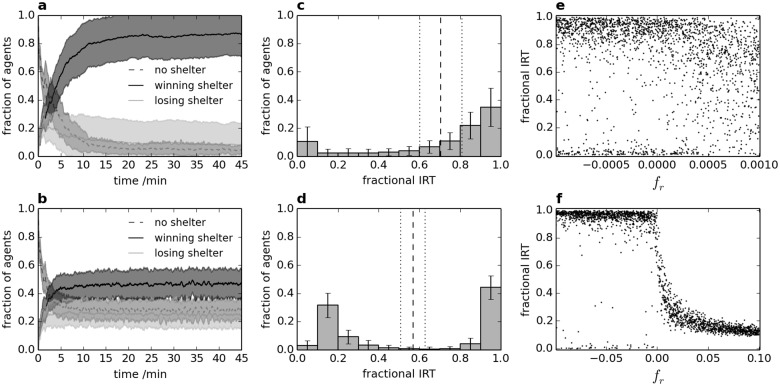
Effect of personality on aggregation and IRT for small and large ranges of randomly distributed *f*_*r*_ values. (a)-(b) Aggregation dynamics for agents with *f*_*r*_ values between ±0.001 and ±0.1 respectively; (c)-(d) corresponding distributions of IRT; (e)-(f) corresponding fractional IRT versus *f*_*r*_, where each dot represents the fractional IRT of a single agent.

In order to investigate the possible role of personality in the aggregation process, the same simulations are run as for Fig 3, but each agent is now given a constant personality value *f*_*r*_ which is assigned randomly for each agent at the start of each simulation. Two sets of simulations are run using a uniform distribution of *f*_*r*_ values, firstly between ±0.001 (termed small range) and secondly between ±0.1 (termed large range); results are shown in Fig 4. As the distributions are symmetrical, the mean value of *f*_*r*_ across all agents is 0 for both personality ranges. A value of *f*_*r*_ > 0 or *f*_*r*_ < 0 makes agent movement more or less likely respectively; please see S3 Fig for consideration of the effects at the group level of uniform (rather than distributed) non-zero *f*_*r*_ values.

The small personality range in Fig 4a, 4c and 4e has little effect on mean IRT or WP compared to Fig 3, but reduces the standard error of IRT from around 0.15 to around 0.1 (note that this measures deviation of the mean value across experiments, rather than the spread of values within experiments). Therefore, although the overall behaviour is similar, it is clearly a more reliable outcome in terms of mean resting time, which may be important for survival. Extremes in behaviour are slightly reduced, with a corresponding increase in intermediate IRT values. Speed of aggregation appears unaffected compared to results for *f*_*r*_ = 0. In contrast, the large personality range in Fig 4 has a far greater effect on group behaviour. Mean IRT, and particularly WP, are both reduced. For WP this is due to the personality of individuals diminishing the social component of behavioural decisions. The reduction in mean IRT is partly due to bold individuals spending more time moving outside shelter (evident in the lower right-hand side of Fig 4f), but also some shy individuals spending more time in aggregates outside shelter (evident in the lower left-hand side of Fig 4f). Importantly, the personality of individuals broadly determines their IRT (as shown by the distinct trend in Fig 4f), rather than random circumstances for uniform personality.

**Fig 5:**
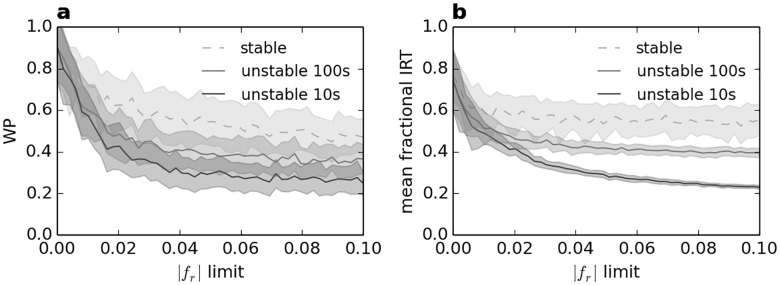
Effect of different magnitudes of *f*_*r*_ range on WP and IRT for stable personality and unstable diversity. Ranges follow a uniform random distribution centred on 0 within the given |*f*_*r*_| limit. Unstable diversity changes the *f*_*r*_ value of agents at regular intervals, as shown in the legend; changes are staggered randomly between agents. (a) WP versus personality range, (b) mean fractional IRT versus personality range.

The consequence of unstable diversity is investigated in order to determine whether the burden of certain behaviours can be shared to provide diversity while minimising costs to individuals. To achieve this, values of *f*_*r*_ are randomly changed at regular time intervals of either 10s or 100s over the entire experimental time of 45 minutes; these changes are randomly staggered between agents. The effect of different *f*_*r*_ ranges on mean IRT and WP are shown in Fig 5, both for stable personality and unstable diversity.

It is evident that both IRT and WP are reduced by the presence of larger magnitudes of *f*_*r*_ values in Fig 5, both for stable personality and unstable diversity. However, unstable diversity has a particularly detrimental effect as it means agents regularly change their propensity to move, thus reducing the stability of aggregates; the unfavourable effect is increased further by shorter time periods between changes. Due to the regular and symmetrically distributed changes of *f*_*r*_, each agent has a mean personality close to *f*_*r*_ = 0 within each experiment. However, the resultant behaviour is very different to the constant value of *f*_*r*_ = 0 in Fig 3: agents with unstable diversity spend far less time under shelter and the consensus decision is much weaker. The variation in IRT is particularly small for unstable personality as this reduces aggregation, which is the main source of diversity in IRT (as aggregation may either be under or outside shelter).

## Discussion and Conclusion

Small differences in personality appear to provide a slight improvement in the consistency of resting time for the group, as shown by comparison of Fig 4c with Fig 3b; the standard error in IRT is reduced from around 0.15 to 0.1 by the presence of personality, without affecting the mean value. However, Fig 4d shows that extremes in personality (with unchanged mean individual behaviour) are detrimental to IRT, due to diminishing the social component of decision making; it is particularly noteworthy that opposing personalities do not balance out at the group level. While the type and magnitude of different personalities are somewhat arbitrary in the present study, results show the importance of all group members responding appropriately to social cues, with important consequences both for the strength of consensus decisions and the ability of individuals to satisfy their own preferences. Results in S3 Fig show the effect of using a multiplicative rather than additive personality factor; this does not diminish the social component of decisions in the same way, but the effect on WP and IRT remains detrimental.

As evident by the pronounced trend in Fig 4f, the personality of an individual is likely to have a large effect on its IRT. Thus, the presence of different personalities is likely to produce a range of behaviours within the group. However, the range of IRT values in Fig 3b shows that even without distinct personalities, diversity of behaviour arises due to the individual circumstances of each agent, which could give a misleading appearance of stable personality; it is therefore important to note that the IRT of an individual in a single experiment is an insufficient measure of personality. In fact, comparison of Figs 3 and 4 shows that diverse individuals may show less variation in behaviour than individuals without differences in personality.

It is clear from Fig 5 that highly diverse personalities are detrimental to consensus decisions (WP) and, to a lesser extent, average resting times (IRT). However, the effect of this is far worse if the diversity is unstable. This demonstrates the importance of stable behaviour both to individuals and the group, which is further to existing understanding of the benefits of personality [23]. Thus stable personality is in itself highly important to promote group stability; this is particularly noteworthy as aggregation is likely to be a pre-requisite for more advanced forms of social behaviour.

The model deliberately uses only a small number of parameters to model aggregation, which are sufficient for the present purposes, but its limitations and potential for further development should be noted. From Fig 3 it is evident that both shelters gain woodlice at a similar rate for a short initial period, but in real life experiments the winning shelter dominates from the outset [5,7], which suggests some form of long-range attraction, herding or initial alignment of woodlice; this requires further investigation. Due to the aims of the study, detailed calibration of the model has not been performed, but this would be necessary in order to simulate particular environmental conditions. For simplicity, the model uses simple movement rules where woodlice effectively follow the arena edge, and dispersal of aggregates is not considered. These details would require development in order to model exploration of different environments, saturation of shelters, and detailed mechanisms to maintain aggregates. Further development of the model would also be required to simulate the spatial distribution of woodlice in nature, including attraction to food sources [29], life-cycle and gender [30], and interactions with other species and the environment [31]. This could be of broad interest, as the study of woodlice aggregation is relevant to a number of areas, including soil dynamics [32], bioindication [33], terrestrialisation and social adaptation [9,34]

Czaczkes et al. [35] recently developed an agent-based model of ant foraging in which individuals specialise in exploiting different resource sites based on their experience of the environment and memory. Given their finding of a tendency of otherwise equal individuals to diversify and specialise, it is possible that some behaviours relating to personality may at least partly reflect the unique experience of each individual (similarly to the results in Fig 3), and could be adaptive. If this is the case, the results in Fig 5 show the imperative for any behavioural changes to be slow.

Different aspects of personality, rather than a simplified representation of shy-bold variation, require further investigation in consensus decisions, as does the effect of learning. Further work is also required to consider the effects of personality on conflicts of interest within the group, as although aggregation may have many benefits, it also has drawbacks [1].

By using an agent-based model of invertebrate aggregation, the effects of personality in consensus decision-making have been investigated. Diverse personalities have only limited benefit within the context of the study, producing a small increase the reliability of consensus decisions, but may have a more valuable role in natural settings. Importantly, while both shy and bold personalities ostensibly have potential downsides at the individual level—resting in the wrong place if shy, not resting enough if bold—all individuals are likely to benefit from maintaining a constant personality in order to promote group stability.

## Supporting Information

S1 Fig Animation of single experiment with *f*_*r*_ = 0 over a simulated time of 5 minutes. Each agent is represented by a circle of radius 2.5mm, distinguished by a random colour; its radial line shows the forward direction. Animation is approximately 5 times faster than simulated time. Shelters are shown in grey. The choice of winning shelter emerges from local interactions between agents. Note that aggregation permits unrealistic overlap between agents since physical dispersal is not modelled, although stacking does occur in real life, as discussed further in the main article.
(MPG)

S2 Fig Effect of a multiplicative personality factor on aggregation and IRT. Rather than adding *f*_*r*_ in Eq 1, here it is instead used as a multiplicative factor. Values for *f*_*r*_ are assigned from a uniform random distribution between ±0.5, and added to 1 to produce the multiplicative factor. (a) Aggregation dynamics; (b) corresponding distribution of IRT; (c) corresponding fractional IRT versus *f*_*r*_,. In comparison with Fig 3 in the main article, both WP and IRT are slightly reduced, but since the social component of decisions is not diminished by this form of personality, behaviour is similar overall.
(PNG)

S3 Fig Effects on IRT of non-zero uniform *f*_*r*_ values. (a) *f*_*r*_ = -0.001, (b) *f*_*r*_ = +0.001, (c) *f*_*r*_ = -0.1, (d) *f*_*r*_ = +0.1. The horizontal line and shaded bar labelled WP show the mean proportion of agents in the winning shelter at the end of the simulation and the standard error. If *f*_*r*_ < 0, movement of agents is decreased, hence aggregation is more likely, which increases the likelihood of extreme IRT values (either small or large depending whether aggregation is outside or under shelter respectively). Conversely, if *f*_*r*_ > 0, movement is increased, hence aggregates are less likely to form, which results in more uniform exploratory behaviour. WP is reduced by non-zero *f*_*r*_ values, as the social component of behavioural decisions is diminished by the increased effect of personal preference. The effect is greater for *f*_*r*_ < 0, where any form of aggregation is less likely. These effects are greater for larger magnitudes of *f*_*r*_, as evident in (c)-(d).
(PNG)
