# Supplementary figures and images for: Simulation of Invertebrate Aggregation Shows the Importance of Stable Personality over Diversity in Consensus Decision-Making
Source: PLoS One. 2016 Oct 18;11(10):e0165082. doi: 10.1371/journal.pone.0165082 (PMC5068703; doi:10.1371/journal.pone.0165082)

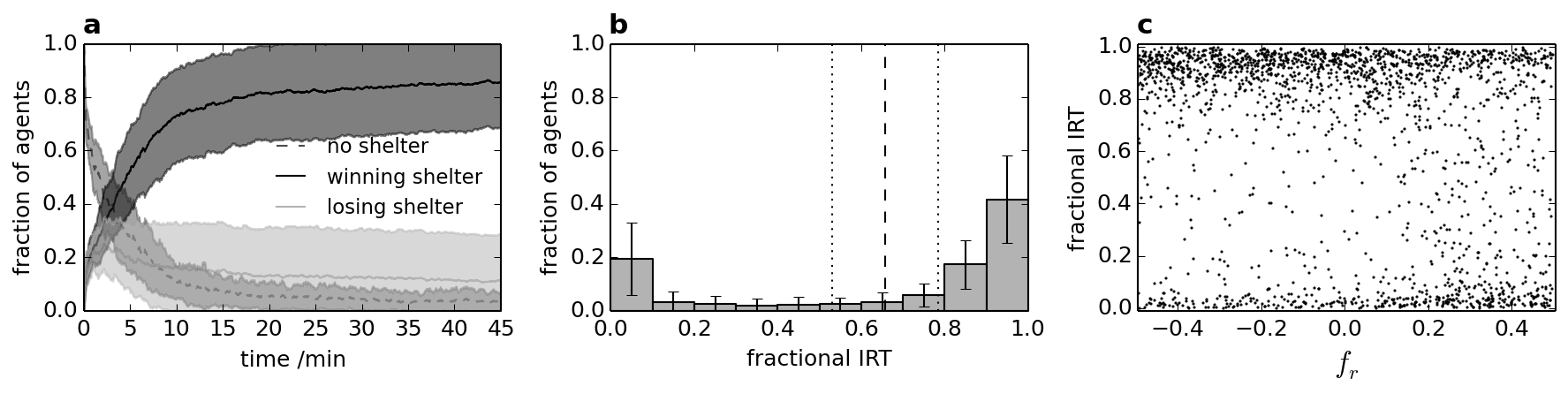

Supplement: S2 Fig — Rather than adding fr in Eq 1, here it is instead used as a multiplicative factor. Values for fr are assigned from a uniform random distribution between ±0.5, and added to 1 to produce the multiplicative factor. (a) Aggregation dynamics; (b) corresponding distribution of IRT; (c) corresponding fractional IRT versus fr,. In comparison with Fig 3 in the main article, both WP and IRT are slightly reduced, but since the social component of decisions is not diminished by this form of personality, behaviour is similar overall. (PNG) [file pone.0165082.s002.png]

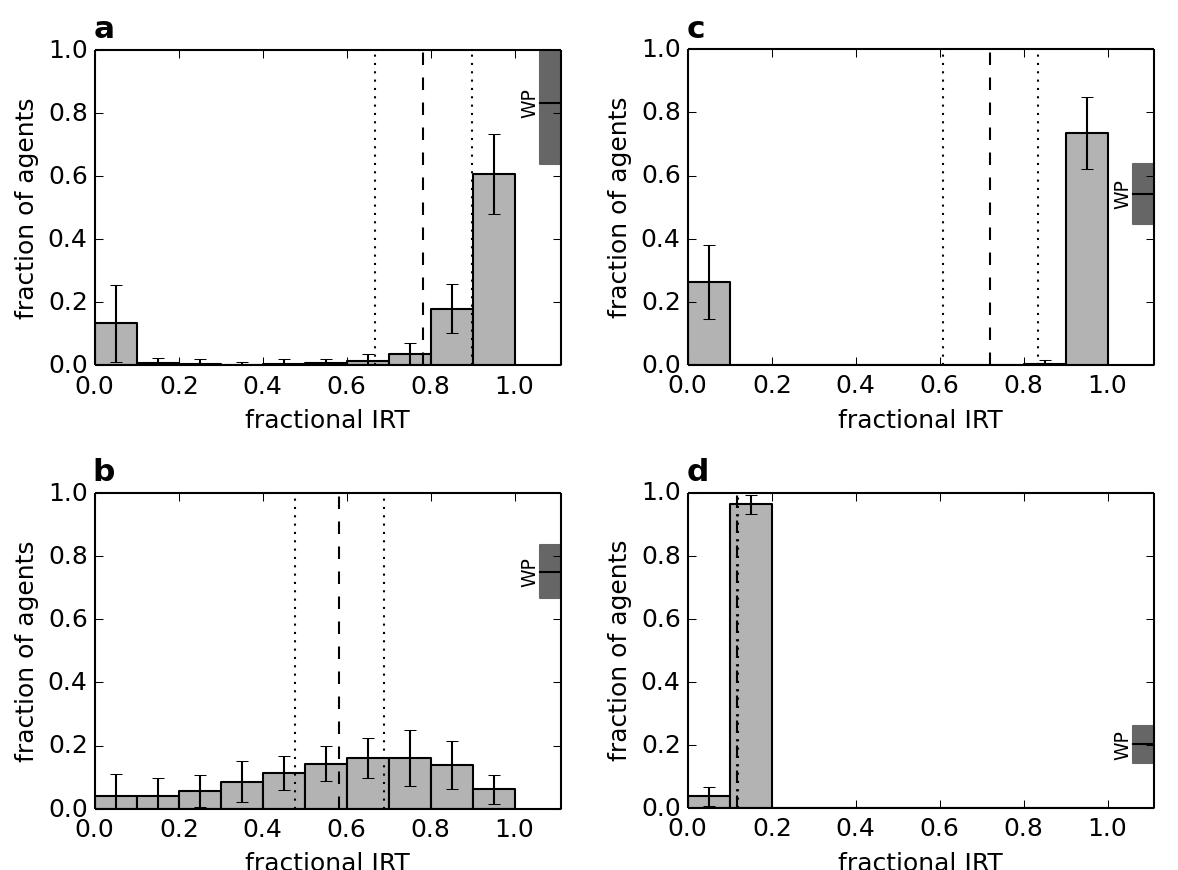

Supplement: S3 Fig — (a) fr = -0.001, (b) fr = +0.001, (c) fr = -0.1, (d) fr = +0.1. The horizontal line and shaded bar labelled WP show the mean proportion of agents in the winning shelter at the end of the simulation and the standard error. If fr < 0, movement of agents is decreased, hence aggregation is more likely, which increases the likelihood of extreme IRT values (either small or large depending whether aggregation is outside or under shelter respectively). Conversely, if fr > 0, movement is increased, hence aggregates are less likely to form, which results in more uniform exploratory behaviour. WP is reduced by non-zero fr values, as the social component of behavioural decisions is diminished by the increased effect of personal preference. The effect is greater for fr < 0, where any form of aggregation is less likely. These effects are greater for larger magnitudes of fr, as evident in (c)-(d). (PNG) [file pone.0165082.s003.png]
